# Medication-related osteonecrosis of the jaw in breast cancer patients: a longitudinal observational Swedish study of oral health and antiresorptive use

**DOI:** 10.1186/s12903-026-09289-0

**Published:** 2026-07-15

**Authors:** Magdalena Korytowska, Gunnar Lengstrand, Anna Ljunggren, Per-Erik Isberg, Cecilia Larsson Wexell

**Affiliations:** 1 Department of Oral and Maxillofacial Surgery and Oral Medicine, Malmö University, Malmö, Sweden; 2 Orofacial Medicine, Folktandvården Region Värmland, Karlstad, Sweden; 3 Department of Oncology, Uddevalla Hospital, Region Västra Götaland, Uddevalla, Sweden; 4 Department of Oral Biology and Oral Pathology, Malmö University, Malmö, Sweden; 5 Department of Statistics, Lund University, Lund, Sweden; 6 Department of Oral and Maxillofacial Surgery, Skåne University Hospital, Lund, Sweden; 7 Department of Clinical Sciences, Lund University, Lund, Sweden

**Keywords:** Antiresorptive therapy, Breast cancer, Breast cancer receptors, Medication-related osteonecrosis of the jaw, Oral health, Survival, Tooth extractions

## Abstract

**Background:**

Breast cancer is one of the most common cancers among women, with improved therapies reducing mortality. Antiresorptive (AR) therapy is crucial for managing bone loss and skeletal complications in patients with cancer. However, high-dose AR therapy may carry a significant risk of medication-related osteonecrosis of the jaw (MRONJ). This study aimed to investigate the incidence of MRONJ and its risk factors in patients with diagnosed breast cancer undergoing either biannual postoperative bisphosphonate therapy or high-dose AR therapy.

**Methods:**

This study followed 220 female patients receiving AR therapy in Sweden, from 2015 to 2020. Oral health status was assessed before and during AR therapy, collecting data on MRONJ incidence, dental procedures, and data on breast cancer characteristics. Statistical analyses included Fisher’s exact tests, and group differences in Kaplan-Meier curves were assessed using the log-rank test. *p* < 0.05 was considered statistically significant.

**Results:**

The cohort comprised 119 patients on biannual AR therapy and 101 patients on high-dose AR therapy. Nine patients (4.0%) developed bone metastases and transitioned to high-dose AR therapy after a mean interval of 237 days; of these, 55.5% subsequently switched from bisphosphonates to denosumab. MRONJ cumulative incidence was 5.5% in the overall cohort and 11.9% in the high-dose group (*p* < 0.001); no MRONJ cases occurred in the biannual group. Tooth extractions (30.5%, *p* < 0.001), localisation to the mandible (83.3%, *p* = 0.039) and number of AR doses (*p* = 0.004) were significantly associated with the development of MRONJ. Notably, spontaneous MRONJ accounted for 41.7% of cases. Baseline DMFT did not differ, but MRONJ patients had a higher DMFT increment during follow-up.

**Conclusions:**

This study identifies a significant association between high-dose AR therapy and the development of MRONJ in breast cancer patients, with tooth extractions, mandibular localisation, and cumulative AR exposure identified as risk factors. MRONJ was not observed in the biannual AR therapy group. These findings underscore the importance of comprehensive dental assessments and early risk stratification.

## Background

Breast cancer remains one of the most prevalent malignancies among women globally. Swedish data reveal a threefold increase in diagnoses between 1960 and 2020 [1], attributed to enhanced screening and detection, an ageing population, lifestyle changes, hormone replacement therapy, and heightened awareness. Despite increasing incidence rates, advances in personalised medicine, immunotherapy, and multimodal treatments have reduced breast cancer annual mortality from 49.0% in 1960 to 13.0% in 2020 [1]. Consequently, a growing population of survivors is now exposed to long-term adverse effects of prolonged oncological treatment.

However, while novel therapeutic approaches have improved survival and overall health outcomes, they have also introduced new challenges for the oral health. Medication-related osteonecrosis of the jaw (MRONJ), a potential complication associated with the use of antiresorptive (AR) agents such as bisphosphonates (BPs) and denosumab (Dmab) [2–4], has been reported worldwide since 2003 [3, 4]. MRONJ may occur following dental procedures such as tooth extractions or may arise spontaneously [2], leading to severe jawbone damage. Patients affected by MRONJ often endure discomfort, pain, and jaw dysfunction, which can severely impair their quality of life [5]. Early identification of MRONJ risk factors, together with timely diagnosis and comprehensive management, is crucial to mitigate these adverse effects [6].

### Biomarkers and personalised treatment in breast cancer

In 2018, the American Joint Committee on Cancer revised its staging manual, emphasising the importance of assessing biological factors to reflect advancements in diagnosis and treatment [7]. Tumour grade, oestrogen receptor (ER), progesterone receptor, and human epidermal growth factor receptor 2 (HER2) status have both predictive and prognostic value and influence treatment selection. This classification was further updated in 2025 [8]. These biomarkers not only guide systemic treatment decisions but also influence long-term treatment exposure, including therapies affecting bone.

### Antiresorptive therapy

Intravenous BPs are routinely administered as postoperative adjuvant therapy to prevent cancer treatment-induced bone loss [9–13] and to reduce the risk of skeletal complications in patients with breast cancer. In the postoperative setting, biannual antiresorptive therapy consisted of intravenous zoledronic acid 4 mg (ZOL) administered every six months for a duration of 3–5 years [1, 11, 14, 15].

In patients with established bone metastases, high-dose AR therapy was administered at monthly intervals, aiming to reduce bone resorption, preserve bone density, and prevent skeletal-related events [1, 2, 16]. High-dose regimens consisted of either intravenous ZOL (4 mg monthly) or subcutaneous Dmab (120 mg monthly), selected according to oncological indication and national clinical guidelines.

### MRONJ incidence

In a Scandinavian post-authorisation safety study, MRONJ among cancer patients treated with AR therapy for solid tumours with metastasis was investigated over a five-year period [17]. MRONJ developed in 1.4% of patients treated with BP (ZOL), 5.7% of patients treated with Dmab and 6.6% of patients who transitioned from ZOL to Dmab [17]. Brunner et al. [18] found similar results in breast cancer patients with bone metastasis, demonstrating that the incidence of MRONJ was 2.8% in patients treated with ZOL, 11.6% in those treated with Dmab, and 16.3% in patients who transitioned from ZOL to Dmab. Patients receiving biannual AR therapy have a low risk of developing MRONJ (≤ 1.5%) [19, 20], and are often managed as low-risk patients [21]. However, current evidence in the published literature remains limited regarding MRONJ risk in patients who initially receive biannual AR therapy and subsequently transition to high-dose AR therapy.

### Preventive oral health management

In Sweden, patients with breast cancer scheduled to receive AR therapy as part of the postoperative medical treatment are referred for a clinical oral evaluation at a specialist clinic for orofacial medicine, to detect and treat odontogenic infections and thereby reduce the risk of developing MRONJ. Among the dental procedures performed, tooth extractions are reported as the major inciting event for MRONJ [2, 22–24]. Despite preventive strategies, the relationship between dental infections, tooth extractions, MRONJ development, breast cancer types, and targeted oncological therapies remains poorly understood [25, 26]. This lack of understanding complicates clinical decision-making, increasing the risk of both undertreatment and overtreatment, which may negatively affect clinical outcomes and quality of life [20, 27, 28]. In particular, there are currently no standardised guidelines for dental management of patients receiving biannual AR therapy who subsequently experience disease progression and require escalation to high-dose AR therapy, creating a clinical challenge in balancing oncological benefit with MRONJ risk [18, 29].

### Aim

The primary aim of this study was to determine the incidence of MRONJ in breast cancer patients undergoing AR therapy. The secondary aim was to explore dental and oncological factors associated with MRONJ development, including oral health status, history of tooth extractions, breast cancer type, antiresorptive agent type, cumulative dosage, and treatment duration, over a six-year longitudinal follow-up.

## Methods

### Study design and participants

This single-centre, longitudinal observational cohort study enrolled patients from the oncology department and the specialist clinic for orofacial medicine at Uddevalla-Trollhättan Hospital, Sweden. Patients who met the inclusion criteria were recruited into the study. These criteria included: women over 18 years of age, a diagnosis of breast cancer, biannual or high-dose treatment with AR agents, and a scheduled oral health examination with any necessary treatment before, during, and after the initiation of AR therapy. Patients were recruited from outpatient populations in Region Västra Götaland. Ethical approval for the study was obtained from the Ethics Review Authority, Sweden (reference numbers 2020 − 00346).

### Data collection

All patients included in the study had undergone breast surgery, either lumpectomy or mastectomy, with axillary sentinel node biopsy and had initiated oncological treatment before referral for oral health evaluation.

#### Oral health evaluation

Clinical examinations, treatment planning, and all dental procedures, including tooth extractions, were performed at a specialist orofacial medicine clinic by six dentists and one dental hygienist, following a standardised protocol. The dentists conducted both the initial oral assessments and the longitudinal management throughout AR therapy. MRONJ cases requiring surgical intervention were referred to the maxillofacial surgery department, while post-operative healing assessments and long-term surveillance were undertaken by the cohort of six dentists at the specialist clinic.

Oral health status was evaluated using the Decayed, Missing, and Filled Teeth (DMFT) index according to World Health Organization criteria [30]. To ensure intra-examiner consistency, all DMFT recordings were performed by a single examiner (the first author), based on the standardised clinical and radiographic documentation generated during routine care. In addition, endodontically treated teeth and apical status were recorded. Periodontal disease was identified through clinical probing and visual inspection, and formal periodontal indexing was not part of the systematic data collection. Additional assessments included the presence and status of prosthetic dentures and bone-anchored implants. Third molars were excluded from the DMFT investigation. Patients were categorised into different age groups for further analysis. Data obtained during the initial visit were collected and compared with data from the final visit.

#### MRONJ

The definition and staging of MRONJ in our study followed the clinical criteria established in the AAOMS 2014 position paper [31]. As these core criteria remain unchanged in the 2022 update [2], the latter was also referenced to reflect current consensus terminology. Data were recorded at the time of clinical suspicion and confirmation of MRONJ, including the treatment modalities employed and treatment outcomes throughout the study period.

MRONJ cases associated with dentoalveolar surgery or other identifiable local risk factors, including apical periodontitis, periodontal disease, and prosthesis-related mucosal trauma, were categorised as odontogenic. Cases without preceding dentoalveolar surgery or documented local trauma at the affected site were classified as spontaneous, although unrecognised mucosal trauma cannot be entirely excluded.

#### Oncological characterisation

During the initial clinical oral examination, data on breast cancer receptor status (ER and HER2), the presence of bone metastases, and details of planned or ongoing oncological treatments were registered for all patients. When available, survival was calculated from the date of referral to the date of death, for both the MRONJ group and the overall cohort.

#### Postoperative medical adjunctive treatment

In our study, endocrine therapy was used for ER-positive tumours, anti-HER2 treatments for HER2-positive tumours, and chemotherapy was administered according to international guidelines [1, 32]. Treatment details were recorded in full only for patients diagnosed with MRONJ.

For all 220 patients, data regarding biannual postoperative BP therapy and high-dose AR therapy regimens, including duration, total number of doses, and transitions between AR therapies, were documented and categorised by age group.

### Statistical analysis

All statistical analyses were performed using IBM SPSS Statistics (Version 27). Fisher’s Exact test was used to compare the incidence of MRONJ between cohort groups. Independent samples t-test was applied to compare AR therapy duration and dosage between patients with MRONJ and the overall cohort, as well as to compare mean DMFT values at the start and at the end of the study. Binomial test was employed to analyse the distribution of MRONJ sites. Patients were stratified into three age categories (30–49, 50–69, and 70–89 years). These categories were defined a priori to ensure adequate subgroup sizes and to facilitate meaningful interpretation of the results. Survival analysis using Kaplan-Meier estimates was conducted across the cohort to assess the time to MRONJ occurrence and the time to death. Group differences in Kaplan-Meier curves were assessed using the log-rank test. A significance level of 5% was applied to all tests.

## Results

The enrolment period was from 1 January 2015 to 31 December 2020. A total of 220 female patients diagnosed with breast cancer, all of whom received AR therapy, were included and underwent oral examination followed by removal of symptomatic pathology. The mean age of the overall population was 65 years (range: 35–88 years; SD = 10.54) (Table 1).

**Table 1:** Demographic and clinical characteristics of the study cohort

	Overall cohort	Biannual treatment	High-dose treatment	
	220 patients	119 patients	101 patients	12 patients (MRONJ)
Age at first appointment				
Years (range)	65 (35–88)	64 (39–85)	67 (35–88)	
Age at first appointment, n (%)				
30–49 years	17 (7.7%)	8 (6.7%)	9 (8.9%)	1 (8.3%)
50–69 years	126 (57.3%)	79 (66.4%)	47 (46.5%)	9 (75.0%)
70–89 years	77 (35.0%)	32 (26.9%)	45 (44.6%)	2 (16.7%)
Breast cancer types, n (%)				
ER+/HER2+	49 (22.3%)	29 (24.4%)	20 (19.8%)	1 (8.3%)
ER+/HER2-	123 (55.9%)	58 (48.7%)	65 (64.3%)	11 (91.7%)
ER+/HER2 (no data)	4 (1.8%)	1 (0.8%)	3 (3.0%)	
ER-/HER2+	15 (6.8%)	12 (10.1%)	3 (3.0%)	
ER-/HER2-	29 (13.2%)	19 (16.0%)	10 (9.9%)	
Skeletal metastases at first appointment, n (%)	92 (41.8%)	0 (0.0%)	92 (91.1%)	11 (91.7%)
Skeletal metastases developed during treatment, n (%)	10 (4.5%)	1 (0.8%)	9 (8.9%)	1 (8.3%)
AR Treatment duration	21.7 months	21.6 months	21.9 months	24.1 months
Days (range)	661 (1-2690)	657 (1-1215)	667 (1-2690)	733 (215–1408)
AR Treatment: Doses Total number (range)	9.5 (1–78)	4.4 (1–6)	15.5 (1–78) *	17.7 (3–39)
AR Treatment: Duration until therapy transition			7.9 months	
Days (range)		-	237 (1-486)	-
AR Treatment: Doses until therapy transition (range)		-	2.4 (1–4)	-
AR treatment until MRONJ diagnosis				23.4 months
Days (range)		-	-	728 (161–1408)

*Nine patients received treatment before first appointment

### Oral health evaluation

Eleven patients wore full dentures at baseline; following dental treatment, this number increased to sixteen, with none observed in the 30–49 age group. Partial dentures were worn across all age groups. No patients in the 30–49 age group had bone-anchored implants. In the 50–69 and 70–89 age groups, a total of forty-seven bone-anchored implants were recorded.

Tooth extraction was the most common procedure during the study period, performed in 30.5% of patients (*n* = 67), including 32 in the biannual group and 35 in the high-dose group. A total of 200 teeth were extracted: 147 by simple extraction and 53 by surgical extraction. Maxillary molars were the most frequently extracted teeth, followed by maxillary premolars and mandibular molars. The indications for extractions were periodontal disease (44.7%) and apical periodontitis (37.9%). In the high-dose group, the first extraction occurred 30 days before AR therapy, and the last extraction was 117 days after AR therapy began. In the biannual group, 72.0% of patients completed all extractions before AR therapy, compared to 60.0% in the high-dose group.

Professional periodontal treatment was provided by a dental hygienist to 56.0% of patients (*n* = 123), including 61 from the biannual group and 62 from the high-dose group.

Fourteen patients (6.4%) received endodontic treatment during the study.

Baseline DMFT scores were comparable between the groups; however, a significant increase in DMFT was observed in 25.0% of MRONJ patients compared to 6.3% of non-MRONJ patients (*p* = 0.047).

### MRONJ

The cumulative incidence of MRONJ was 5.5% (12/220) in the overall cohort and 11.9% (12/101) among patients receiving high-dose AR therapy. No MRONJ cases were observed in the biannual AR therapy group.

Statistical analysis showed a significant association between MRONJ and tooth extractions (*p* < 0.001), but no significant correlation with periodontal therapy (*p* = 0.235) or endodontic therapy (*p* = 0.172).

MRONJ was identified at a mean of 11 months after tooth extraction at the affected site. In contrast, spontaneous MRONJ onset occurred in 41.7% (5/12) of the cases, on average, 30 months after the initiation of AR therapy. For the rest of the MRONJ patients (6/12), tooth extractions were completed before starting AR treatment. The mean time from initiation of high-dose AR therapy to MRONJ onset was not significantly different from the overall cohort’s mean (*p* = 0.579). The mean number of AR doses in MRONJ patients was 18 (range: 3–39), which was significantly higher than the cohort’s mean of 9 doses (*p* = 0.004) until the end of the study.

Among the 12 patients diagnosed with MRONJ (Table 2), 6 (50.0%) had infection-associated disease, including 3 cases related to apical periodontitis and 3 to periodontal disease. In 5 of these cases, tooth extraction was performed due to infection. Prosthesis-related mucosal trauma associated with an ill-fitting denture was identified in 1 patient (8.4%). In the remaining 5 cases (41.7%), no prior tooth extraction or surgical intervention at the affected site was documented; these cases were classified as spontaneous.

**Table 2:** Baseline demographic and clinical characteristics of patients with MRONJ

Patient	Receptor type	AR treatment	Duration of AR therapy until MRONJ (days)	MRONJ stage at diagnosis	MRONJ stage at the study’s completion	MRONJ clinical history	MRONJ location (region)	MRONJ treatment
1	ER+/HER2-	ZOL+Dmab	(113 + 512) 625	2	1	Periodontal disease	Mandible (31–41)	AR therapy discontinuation Antibiotic treatment (phenoxymethylpenicillin) Surgical treatment
2	ER+/HER2-	ZOL+Dmab	(230 + 1178) 1408	2	1	Spontaneous (non-extraction)	Mandible (34–36)	AR therapy discontinuation Antibiotic treatment (phenoxymethylpenicillin, phenoxymethylpenicillin + metronidazole)
3	ER+/HER2-	ZOL	1372	2	3	Spontaneous (non-extraction)	Mandible (34–36)	AR therapy discontinuation Antibiotic treatment (phenoxymethylpenicillin, phenoxymethylpenicillin + metronidazole, amoxicillin) Surgical treatment
4	ER+/HER2-	ZOL	263	1	1	Periodontal disease / Tooth Extraction	Mandible (46)	AR therapy discontinuation Antibiotic treatment (clindamycin)
5	ER+/HER2-	Dmab	422	1	resolved MRONJ	Periodontal disease / Tooth Extraction	Mandible (31–41)	AR therapy discontinuation Antibiotic treatment (phenoxymethylpenicillin) Surgical treatment
6	ER+/HER2-	Dmab	477	1	resolved MRONJ	Periodontal disease /Tooth extraction /ill-fitting prostheses	Maxilla (16,14,24,25)	AR therapy discontinuation* Surgical treatment
7	ER+/HER2-	ZOL	215	1	1	Spontaneous (non-extraction)	Mandible (44–45)	AR therapy discontinuation
8	ER+/HER2-	Dmab	433	1	3	Periapical disease /Tooth extraction	Mandible (45)	AR therapy discontinuation Antibiotic treatment (clindamycin + metronidazole)
9	ER+/HER2-	ZOL	1273	2	1	Periapical disease /Tooth extraction	Mandible (37)	AR therapy discontinuation Antibiotic treatment (amoxicillin/clavulanic acid)
10	ER+/HER2-	ZOL	161	1	resolved MRONJ	Spontaneous (non-extraction)	Mandible (47)	AR therapy discontinuation* Chlorhexidine rinse Antibiotic treatment (clindamycin)
11	ER+/HER2+	ZOL+Dmab	(70 + 1059) 1129	1	resolved MRONJ	Spontaneous (non-extraction)	Mandible (48)	AR therapy discontinuation Chlorhexidine rinse Antibiotic treatment (phenoxymethylpenicillin)
12	ER+/HER2-	ZOL+Dmab	(63 + 390) 453	3	resolved MRONJ	Periodontal disease / Tooth extraction	Maxilla (16)	AR therapy discontinuation Antibiotic treatment (phenoxymethylpenicillin, clindamycin, amoxicillin) Surgical treatment

* Antiresorptive therapy resumed after documented MRONJ resolution

The mandible was the most affected site in MRONJ, accounting for 83.3% of cases (10/12; *p* = 0.039).

**Fig. 1:**
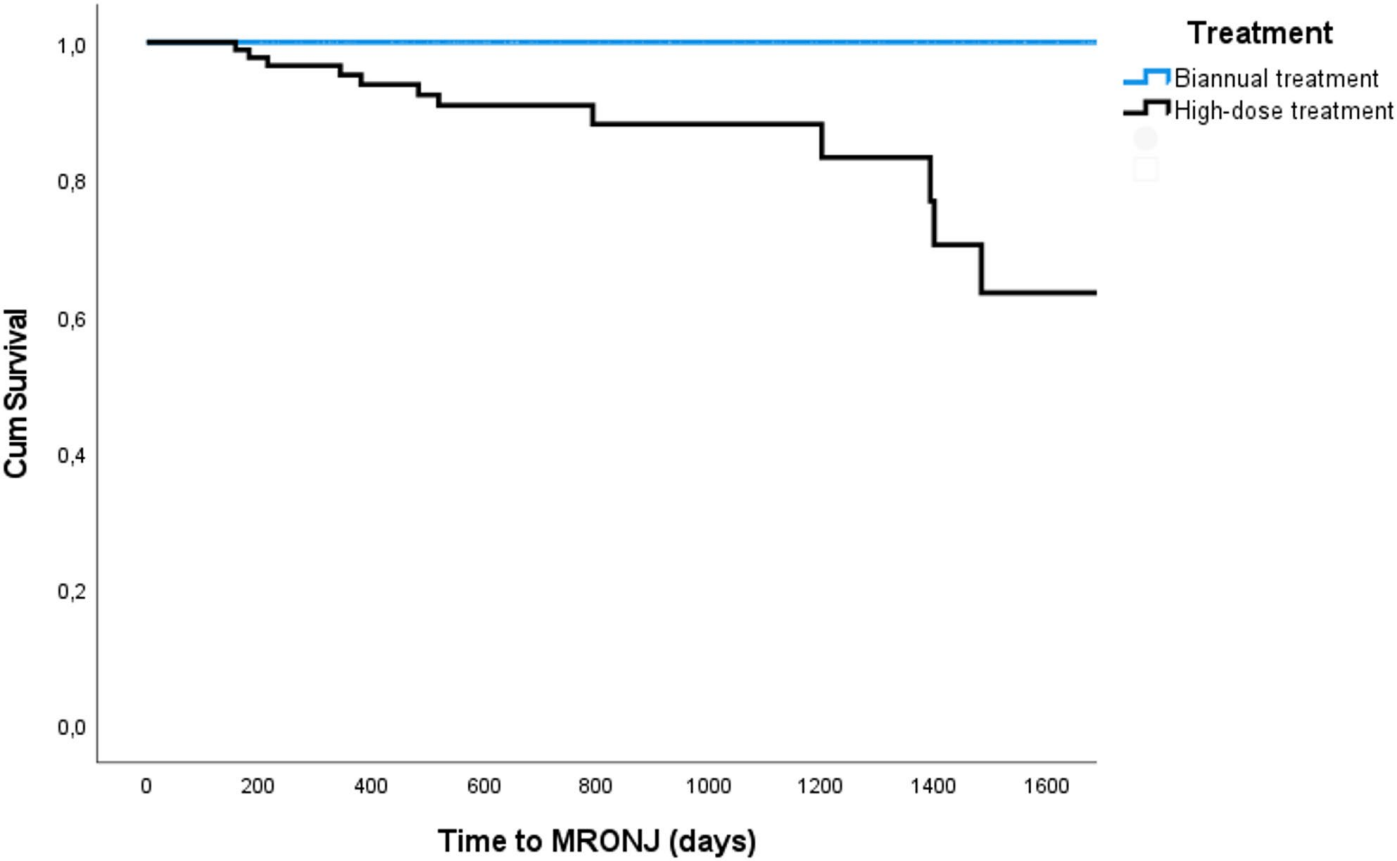
Kaplan-Meier analysis of time to MRONJ by antiresorptive treatment; log-rank test, *p* < 0.001

**Fig. 2:**
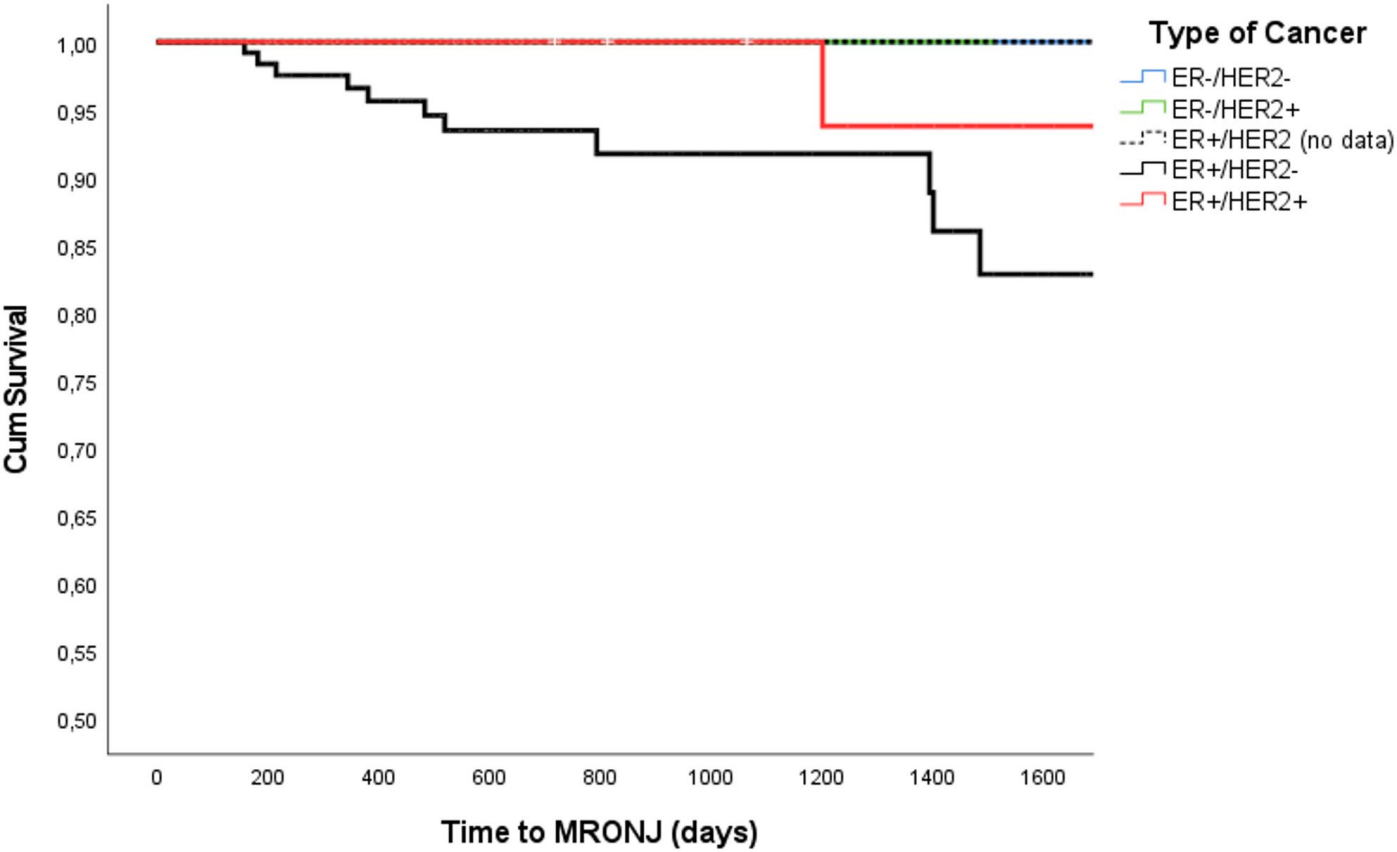
Kaplan-Meier analysis of time to MRONJ by receptor type; log-rank test, *p* = 0.179

**Fig. 3:**
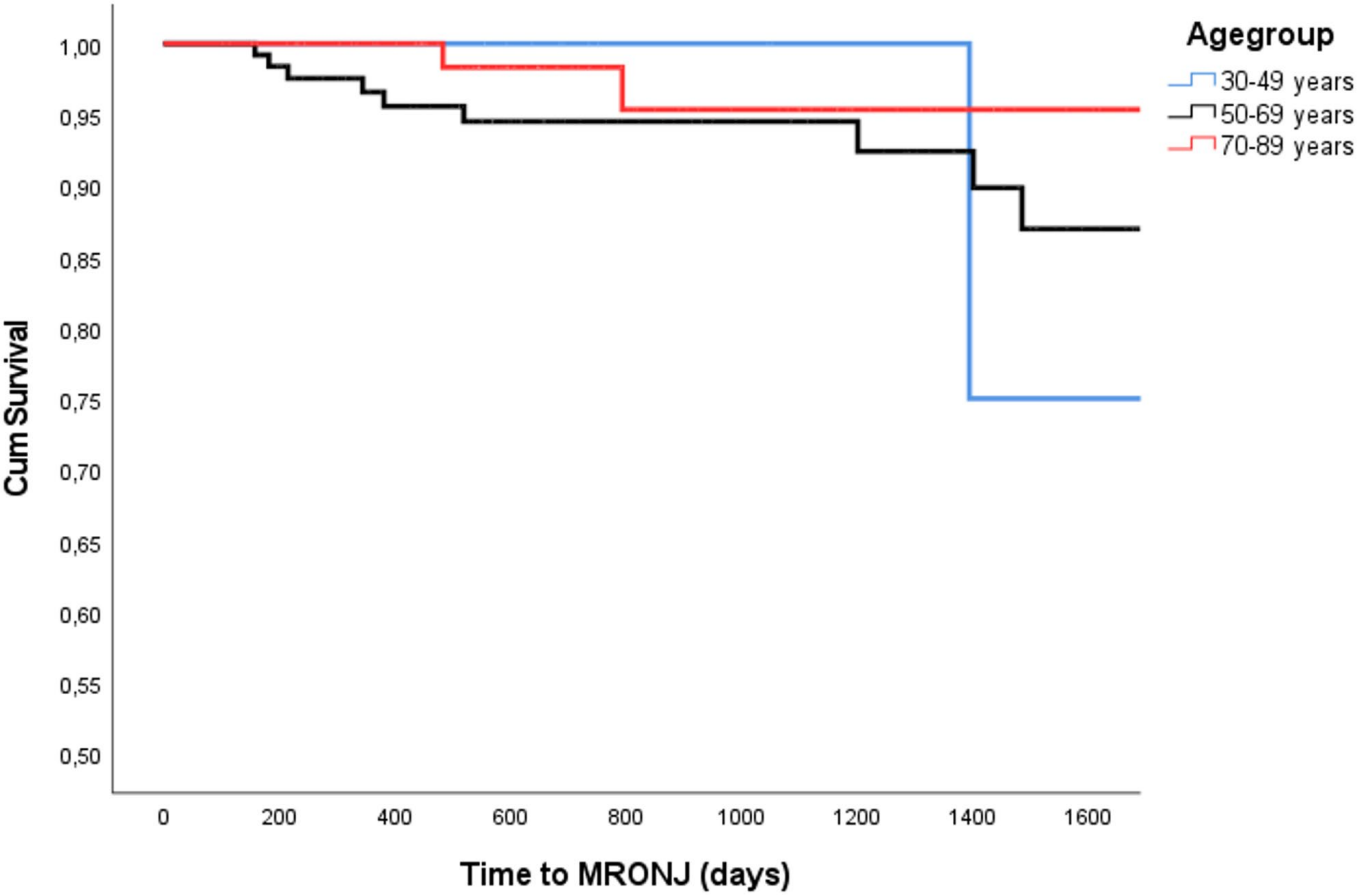
Kaplan-Meier analysis of time to MRONJ by age group; log-rank test, *p* = 0.687

MRONJ was diagnosed in patients receiving high-dose AR therapy (*p* < 0.001). All affected patients had ER-positive breast cancer (100.0%, *n* = 12), with the majority (91.6%, *n* = 11) classified as HER2-negative, but no significant association was found between breast cancer type and MRONJ development (*p* = 0.157). Similarly, age did not significantly correlate with MRONJ (*p* = 0.311), although most cases (75.0%, *n* = 9) occurred in patients aged 50–69. Kaplan-Meier analysis demonstrated a significant difference in time to MRONJ between treatment groups (log-rank test, *p* < 0.001), whereas no statistically significant differences were observed by receptor type (log-rank test, *p* = 0.179) or age group (log-rank test, *p* = 0.687) (Figs. 1, 2 and 3).

The type of AR drug used was not a significant factor in MRONJ development (*p* = 0.056). Among affected patients, 41.7% (5/12) received ZOL and 25.0% (3/12) received Dmab. Transitions between AR medications (ZOL-Dmab) were found for 33.3% (4/12) of cases (Table 3).

**Table 3:** Antiresorptive agent types in the study cohort

Antiresorptive drug	Overall cohort *n* = 220	Biannual group *n* = 119	High-dose group *n* = 101	MRONJ group *n* = 12
Zoledronic acid 4 mg (ZOL)	146 (66.4%)	119 (100.0%)	28 (26.7%)	5 (41.7%)
Denosumab 120 mg (Dmab)	44 (20.0%)		44 (43.6%)	3 (25.0%)
Transition: ZOL to Dmab	26 (11.8%)		25 (25.7%)	4 (33.3%)
Transition: Dmab to ZOL	3 (1.4%)		3 (3.0%)	
Transition: ZOL to Dmab to ZOL	1 (0.5%)		1 (1.0%)	

* Categorisation reflects the final AR treatment received

No statistically significant associations were observed between MRONJ occurrence and age, periodontal disease, or breast cancer type; however, these analyses were based on a limited number of MRONJ events resulting in low statistical power.

### Oncological evaluation

Overall, 80.0% of patients (*n* = 176) were ER-positive, and 72.0% (*n* = 152) were HER2-negative. The most common type was ER+/HER2-, accounting for 56.0% (*n* = 123) of the cohort. Patients with ER-negative and HER2-negative type had lower five-year survival compared to those with ER+ type. In our study, patients aged 50–69 years (*n* = 126) had higher five-year survival rates compared with both younger and older patients.

#### Postoperative medical adjunctive treatment – MRONJ group

Medication data for MRONJ patients were limited due to incomplete records for 8 of the 12 patients, whose breast cancer diagnoses ranged from 1979 to 2012. Consequently, only the names of medications, including chemotherapy and endocrine therapy, are provided, without information on dosages or treatment durations. Tumour recurrence in 5 patients between 2003 and 2019 introduced additional complexity.

The therapeutic regimens of the 12 MRONJ patients were categorised based on chemotherapy (anthracyclines, taxanes) and endocrine therapy (aromatase inhibitors, antioestrogens), demonstrating significant treatment variability (Table 4). Patients 4 and 7 received multi-drug regimens combining anthracyclines, taxanes, aromatase inhibitors, and antioestrogens. Limited information was available for patient 6. Aromatase inhibitors were prescribed to all patients, and 8 out of 12 received antioestrogens. Postoperative medical treatment (antineoplastic chemotherapy/anthracyclines) data were missing for patients 1, 5, 8, 9, and 10.

**Table 4:** Postoperative adjunctive treatments in patients with MRONJ

Patient	Antineoplastic chemotherapy/ Anthracyclines	Antineoplastic chemotherapy/ Taxanes	Endocrine therapy/ Aromatase inhibitors	Endocrine therapy/ Antioestrogens
1	data not available	data not available	exemestane	0
2	0	paclitaxel	letrozole exemestane	tamoxifen
3	epirubicin	paclitaxel	letrozole	0
4	epirubicin	docetaxel	anastrozole	tamoxifen
5	data not available	data not available	letrozole	tamoxifen
6	0	paclitaxel	letrozole	
7	epirubicin	paclitaxel	anastrozole	tamoxifen
8	data not available	data not available	letrozole	tamoxifen
9	data not available	data not available	letrozole	tamoxifen
10	data not available	data not available	no data	
11	0	paclitaxel	letrozole	tamoxifen fulvestrant
12	0	paclitaxel	letrozole	fulvestrant

#### Postoperative antiresorptive therapy in the cohort

At baseline, 128 patients were assigned to the biannual group and 92 to the high-dose group. Disease progression resulted in bone metastases in nine patients (4.0%) within the biannual group, who were subsequently transitioned to high-dose AR therapy. This reclassification resulted in a final cohort of 119 in the biannual group and 101 in the high-dose group. An overview of AR drug type, dosage, administration interval, and treatment duration for the study cohort is provided in Table 1.

All patients in the biannual group received ZOL. In the high-dose group, Dmab was the most prescribed agent (43.6%), followed by ZOL (26.7%) (Table 3).

Among the 9 patients with disease progression, 5 transitioned from biannual ZOL to monthly Dmab injections, while 4 continued ZOL infusions, but monthly. The total duration of AR therapy in this group was 541 days (17.8 months), with a mean of 9 doses per patient (range: 3–20). Changes in the AR regimen occurred after an average of 237 days (7.9 months).

#### Survival

During follow-up, mortality was 5.9% (7/119) in the biannual AR group and 51.5% (52/101) in the high-dose group, while 41.7% (5/12) of patients with MRONJ died (Table 5). The mean time to death was 24.3, 21.8, and 23.4 months, respectively. Mortality in the biannual group was evenly distributed between patients aged 50–69 and 70–89 years, whereas in the high-dose group it was highest among those aged 70–89 years. In the MRONJ cohort, most deaths occurred in patients aged 50–69 years.

**Table 5:** All-cause mortality in the study cohort during follow-up

	Biannual treatment 119 patients	High-dose treatment 101 patients	MRONJ 12 patients
Deaths during follow-up, *n* (%)	7 (5.9%)	52 (51.5%)	5 (41.7%)
Mean time to death,	24.3 months	21.8 months	23.4 months
days (range)	738 (310–1081)	662 (112–1941)	712 (244–1941)
Mortality and breast cancer types, n (%)			
ER+/HER2+	1 (14.3%)	15 (28.8%)	
ER+/HER2-	2 (28.6%)	25 (48.1%)	5 (100.0%)
ER+/HER2 (no data)	1 (14.3%)	3 (5.8%)	
ER-/HER2+	0 (0.0%)	1 (1.9%)	
ER-/HER2-	3 (42.9%)	8 (15.4%)	
Mortality and Age group, n (%)			
30–49 years	1 (14.3%)	5 (9.6%)	1 (20.0%)
50–69 years	3 (42.9%)	19 (36.5%)	4 (80.0%)
70–89 years	3 (42.9%)	28 (53.8%)	0 (0.0%)

*Percentages are calculated within the number of deaths in each treatment group and may not sum to 100.0% due to rounding

## Discussion

To our knowledge, this is the largest Swedish study to date investigating oral health and MRONJ development in breast cancer patients treated with AR agents, administered either biannually or at high-dose, over a six-year period. Retrospective data collection commenced in 2015, shortly after the national implementation of routine postoperative AR therapy for breast cancer patients without bone metastasis, administered at biannual intervals. The study concluded in late 2020, when treatment protocols were modified in response to the onset of the COVID-19 pandemic.

The risk of MRONJ in patients receiving AR therapy has been reported in the literature; however, significant uncertainty remains regarding the influence of oral health, dental diagnoses and procedures, breast cancer types, and biannual administration of BPs on MRONJ development. Although previous studies have independently investigated the association between AR therapy [2, 33] and the occurrence of MRONJ, comprehensive research addressing the combined influence of breast cancer receptor types, such as ER and HER2, their related oncological treatments, and oral health status on MRONJ risk remains limited.

Our findings highlight clinically relevant differences in MRONJ risk between biannual and high-dose AR regimens. All patients were managed according to a standardised protocol, reflecting current clinical practice in the absence of stratified guidelines. This protocol encompassed infection management (tooth extractions, periodontal treatment, root canal therapy, and caries management) and the provision of removable dentures for aesthetic purposes.

The oral health assessments and DMFT index observed in our study were consistent with those of the general Swedish population [34], supporting the generalisability of our findings. However, MRONJ patients exhibited a greater increase in DMFT over time, suggesting a higher burden of dental complications. Despite this, baseline oral health parameters, including the DMFT index, were comparable between patients with and without MRONJ, indicating that factors arising during follow-up, such as progressive periodontal disease or tooth fractures requiring extraction, may increase MRONJ risk.

Non-surgical periodontal and endodontic treatments were not associated with MRONJ development. The absence of statistically significant associations between MRONJ and factors such as age, periodontal disease, or breast cancer type should be interpreted with caution. Given the low number of MRONJ cases in the present cohort, the study was likely underpowered to detect modest to moderate risk effects. Consequently, the lack of statistical significance may reflect a Type II error rather than a true absence of association.

MRONJ occurred predominantly in the mandible, consistent with previous reports, likely due to the denser bone structure of the mandible, the greater complexity of extraction procedures, and their potential impact on postoperative healing, which may increase susceptibility to MRONJ [21, 29, 35].

In the present cohort, surgical and conservative management resulted in complete mucosal healing in 5 of 12 patients (41.7%) by the end of follow-up. An additional 3 patients (25.0%) demonstrated clinical improvement with downstaging of MRONJ, whereas 2 patients (16.7%) remained stable and 2 (16.7%) progressed despite surgical intervention. Although the number of cases is limited, these outcomes are broadly consistent with previously published studies reporting healing or improvement rates ranging between approximately 60–85% following stage-appropriate surgical management [2, 21, 36, 37]. Variability in outcomes is likely influenced by disease stage at diagnosis, cumulative antiresorptive exposure, and systemic oncological factors. Our findings support the role of individualised surgical intervention within a multidisciplinary framework, while highlighting that progression may still occur in a subset of patients despite treatment.

In a review by Ng et al. [33], cancer patients exhibited a higher MRONJ risk after prolonged AR exposure. Consistent with this, in our study, the average time between tooth extractions and MRONJ onset was 11.4 months.

Spontaneous (non-extraction related) MRONJ accounted for a substantial proportion of cases in this study, comparable to the findings of Mauceri et al. [29]. This finding highlights that MRONJ may develop independently of surgical triggers and underscores the importance of continued oral surveillance in high-risk patients.

Our results show that 80.0% of the patients were ER-positive, of whom 56.0% were classified as HER2-negative, in alignment with established epidemiology. Ramchand et al. [12] highlight that aromatase inhibitors increase bone loss and fracture risk, whereas antioestrogens exert differential effects depending on menopausal status. Given that most MRONJ patients received prolonged endocrine therapy, treatment-related bone vulnerability may have contributed to MRONJ susceptibility. Although ER-positive disease predominated among MRONJ cases, this association did not reach statistical significance and should be regarded as hypothesis-generating rather than confirmatory.

In the present cohort, the overall cumulative incidence of MRONJ was 5.5%, while the cumulative incidence in the high-dose group was 11.9%, aligning with previously reported rates in metastatic breast cancer cohorts. AR therapy is a well-known risk factor for MRONJ development, with a cumulative risk of less than 5.0% (range: 0.0–18.0%) in cancer patients treated with high-dose BPs (e.g., ZOL) [2]. Patients receiving Dmab exhibit a risk ranging from 0.0 to 6.9%, although studies report rates below 5.0% [2]. In this cohort, MRONJ occurred exclusively in patients receiving high-dose AR therapy, supporting a dose-response relationship. Cumulative AR exposure emerged as a key risk factor, consistent with previous studies [2, 33].

The risk of MRONJ development has also been studied in non-metastatic breast cancer patients receiving biannual BP therapy [14, 16, 19–21, 38]. In the AZURE study, which included 3,360 postmenopausal women with early-stage breast cancer and a median follow-up of approximately 10 years, treatment with ZOL was associated with significant improvement in both disease-free survival and invasive disease-free survival [15]. MRONJ occurred in 1.8% of the patients. Patel et al. [19] reported MRONJ incidences ranging from 0.0% to 1.5%, while Kizub et al. observed a 1.26% incidence (28/2,231 patients) [20]. Mauceri et al. [21] identified 15 patients with biannual AR treatment with a mean duration of MRONJ onset at 37 months (*±* 26.3 months), of whom 60.0% were treated with aromatase inhibitors. Despite low overall incidence rates, these studies underscore the small but not insignificant rate of MRONJ. With regard to risk factors, pre-treatment dental evaluations and the use of alert cards have been suggested to support MRONJ prevention [19], as poor oral health (periodontitis, extractions, and trauma) has been identified as a potential contributor to MRONJ development [20], although this was not confirmed in our study.

In our cohort, no cases of MRONJ were observed among patients receiving biannual BP therapy during the follow-up period. This corresponded to a cumulative incidence of 5.5% overall (9.0 cases per 1,000 patient-years) and 11.9% in the high-dose group (*p* < 0.001). The observed association between high-dose AR therapy and MRONJ should be interpreted in the context of underlying disease severity, as patients in the high-dose group predominantly had bone metastases and greater systemic treatment exposure. Kaplan-Meier time-to-event analysis confirmed that all MRONJ events occurred in the high-dose group. However, this finding should be interpreted with caution. The absence of observed cases in the biannual group does not necessarily imply absence of risk, as the limited number of events and the duration of follow-up may not fully capture late-onset MRONJ. Although follow-up periods overlapped between treatment groups, longer-term observation in larger cohorts is required to determine whether the difference reflects treatment intensity or cumulative exposure over time.

No statistically significant association between AR drug type and MRONJ development was observed in this cohort. However, the borderline result (*p* = 0.056) should be interpreted cautiously, as it likely reflects insufficient statistical power due to the low number of MRONJ events. This finding should therefore be considered hypothesis-generating and warrants further investigation in larger cohorts.

Our findings did not demonstrate an association between transitions in treatment regimen and the development of MRONJ (*p* = 0.056). However, a substantial proportion of MRONJ cases occurred following transitions from ZOL to Dmab. In a prospective study by Ehrenstein et al. [17], almost 2900 cancer patients with solid tumours and metastases treated with either ZOL or Dmab were examined with expert MRONJ adjudication. Over the course of 5 years, MRONJ developed in 1.4% of the patients treated with ZOL, in 5.7% of the patients treated with Dmab and in 6.6% of the patients who switched from ZOL to Dmab. Switching from BP to Dmab has been hypothesised not only to reflect a longer treatment duration, but also to serve as an independent risk factor for MRONJ [39–41]. In a retrospective cohort of 14 breast cancer patients, Mauceri et al. [29] reported that 28.6% (*n* = 4) transitioned to high-dose AR therapy, of whom 50.0% subsequently developed MRONJ. Similarly, Brunner et al. [18] reported an MRONJ incidence of 8.8% over 20 years and highlighted an increased risk associated with transitions in AR therapy (16.3%), emphasising the need for careful management during such transitions. Notably, the median time to MRONJ onset was 8.4 years from the initiation of AR therapy in patients who transitioned therapies (BP to Dmab), compared with 4.6 years in those receiving Dmab alone.

In line with previous studies [29], our findings further underscore the need for a comprehensive approach to MRONJ prevention in breast cancer patients receiving AR therapy, regardless of dose intensity. Preventive strategies should include both pre-treatment dental optimisation and ongoing surveillance, particularly in patients receiving high-dose or transitioning AR regimens. Given the multifactorial pathogenesis of MRONJ, close multidisciplinary collaboration remains essential.

## Conclusion

Antiresorptive therapy plays a crucial role in reducing cancer treatment–induced bone loss and preventing skeletal-related events in breast cancer patients. However, higher cumulative exposure may increase the risk of adverse effects, including MRONJ.

In this cohort, MRONJ was significantly associated with high-dose AR therapy, cumulative treatment duration and dose, and tooth extractions. Spontaneous MRONJ also accounted for a substantial proportion of cases. While periodontal and endodontic treatments showed no independent association with MRONJ development, tooth extractions emerged as the primary precipitating factor. Although no statistically significant association was observed between breast cancer type and MRONJ, ER+/HER2 − disease predominated among affected patients and should therefore be interpreted as hypothesis-generating. These findings support careful risk assessment, minimally traumatic surgical approaches, and multidisciplinary management, particularly in patients receiving high-dose AR therapy or transitioning between regimens.

Future research should focus on the refinement of multifactorial risk stratification models and the optimisation of preventive protocols to enhance long-term clinical outcomes.

### Limitations

The longitudinal design and comprehensive oral health assessments strengthen the validity of our findings. However, the conclusions are limited by the small cohort size and the low number of patients diagnosed with MRONJ. In addition, patients were enrolled over a six-year period, resulting in variability in follow-up. A further limitation is the lack of systematically recorded periodontal indices; although examinations followed a standardised protocol, inter-examiner variability cannot be excluded. The small number of MRONJ events limits the statistical power and increases the risk of Type II error, particularly in analyses of demographic, dental, and oncological factors. All MRONJ cases occurred in the high-dose AR therapy group, which largely comprised patients with bone metastases. This introduces potential confounding by disease severity, metastatic burden, and greater systemic treatment exposure. Due to the limited number of events, multivariable adjustment was not feasible, and the independent effect of high-dose AR therapy on MRONJ risk cannot be separated from that of underlying metastatic disease. In addition, group comparisons using log-rank tests are underpowered and should therefore be interpreted with caution.

## Data Availability

The dataset generated and analysed during the present study is available from the corresponding author upon reasonable request.

## Abbreviations

AR: Antiresorptive
BP: Bisphosphonate
Dmab: Denosumab
DMFT: Decayed, Missing, and Filled Teeth
MRONJ: Medication-related osteonecrosis of the jaw
ER: Oestrogen receptor
HER2: Epidermal growth factor receptor 2
ZOL: Zoledronic acid
